# Dr. Delos Burd Manchester

**Published:** 1912-09

**Authors:** 


Dr. Delos Burd Manchester, died at Schenevus, N. Y., July 21, aged 54. He was a graduate of Buffalo, 1883. For many years he practiced at Oneonta, N. Y., later at Grant's Pass, Ore.

